# Predictors of Postoperative Pneumonia Following Anatomical Lung Resections in Thoracic Surgery

**DOI:** 10.3390/jcm14238445

**Published:** 2025-11-28

**Authors:** Timon Marvin Schnabel, Kim Karen Kutun, Martin Linde, Jerome Defosse, Mark Ulrich Gerbershagen

**Affiliations:** 1 Department of Anaesthesiology, Cologne-Holweide Hospital, University Witten/Herdecke, Neufelder Str. 32, 51067 Cologne, Germany; 2 Department of Anaesthesiology and Intensive Care Medicine, Cologne-Merheim Hospital, University Witten/Herdecke, Ostmerheimer Strasse 200, 51109 Cologne, Germany

**Keywords:** anatomical lung resection, perioperative complications, postoperative pneumonia, postoperative ventilation, risk factors, thoracic surgery

## Abstract

**Background/Objectives:** Postoperative pneumonia (PP) is a significant complication following thoracic surgery, increasing morbidity, mortality, and hospital length of stay. Identifying risk factors is crucial for optimizing perioperative management. This study analyses predictors for PP in patients undergoing anatomical lung resections in a single center setting. **Methods:** A prospective cohort study was conducted using data from the German Thoracic Registry (GTR). Patients who underwent anatomical lung resection were included in the study, while non-anatomical resections and cases with missing data were excluded. The primary outcome measure was the incidence of PP, which was analyzed using chi-square tests and Fisher’s exact test. **Results:** PP was observed in 15.2% of the 381 patients. Significant preoperative predictors included American Society of Anesthesiologists (ASA) classification ≥ 3 (*p* = 0.021), C-reactive protein (CRP) ≥ 20 mg/L (*p* = 0.004), white blood cell count (WBC) ≥ 15,000/µL (*p* = 0.003) and forced expiratory volume in 1 s (FEV1) < 50% (*p* = 0.004). Intraoperative risk factors included thoracotomy (THT) (*p* = 0.001) and duration of operation > 180 min (*p* = 0.002). Postoperative predictors included Intensive Care Unit (ICU) admission (*p* < 0.001) and mechanical ventilation > 24 h (*p* < 0.001). PP was associated with a higher perioperative mortality rate (10.3% vs. 1.2%, *p* = 0.01) and prolonged hospital stay. **Conclusions:** A number of risk factors for the development of PP have been identified, which may help to reduce the incidence of the condition. For further validation, multicenter studies are required.

## 1. Introduction

The objective of this study was to ascertain the risk factors associated with the development of Postoperative Pneumonia (PP) in patients undergoing anatomical lung resection at a single center.

PP is a prevalent complication, with a significant increase in mortality and prolonged hospitalization rates [1,2]. The extant literature suggests a prevalence of 5–15% of PP in patients undergoing thoracic surgery [2], with an increased mortality when compared with patients who do not experience PP [3]. PP is also associated with longer hospital stays and higher discharge to facility rates [1,4].

The present study specifically addresses this gap by prospectively analyzing a single-center cohort of anatomical lung resections with standardized definitions, focusing on actionable inflammatory thresholds (CRP ≥ 20 mg/L; WBC ≥ 15,000/µL), surgical approach (VATS vs. thoracotomy) and operative duration, with the aim of informing practical perioperative decision-making.

Thoracic surgery, including pneumonectomy, bilobectomy, lobectomy and segmentectomy, is a fundamental component of contemporary lung cancer therapy [5]. In this context, effective surgical intervention is crucial for patient survival [6]. Recent studies indicate that thoracic surgery has become increasingly important in the treatment of lung cancer [7]. The early identification of risk factors can also improve perioperative patient safety, reduce morbidity and decrease mortality rates [8].

The issue of PP is twofold in nature. Firstly, it has been demonstrated to have a detrimental effect on clinical outcomes. Secondly, it has been shown to impose a significant economic burden on patients, which is due to the extended duration of hospitalization and the increase in healthcare costs [1,9,10]. Several studies have identified various risk factors as critical determinants of postoperative complications in thoracic surgery, particularly PP. These include patient characteristics such as advanced age, male gender, a history of smoking, and alcohol abuse, which have been shown to significantly contribute to the risk. In addition, health status indicators such as a high American Society of Anesthesiologists classification (ASA) ≥ 3, the presence of Chronic Obstructive Pulmonary Disease (COPD), and compromised lung function (as evidenced by reduced Forced expiratory volume in one second (FEV1), FEV1/Forced vital capacity (FVC), and Diffusion Capacity of the Lung for Carbon monoxide (DLCO)) further exacerbate this risk [1,4,11]. Furthermore, inflammatory and treatment-related markers, such as elevated C-Reactive Protein (CRP) [12], previous chemotherapy or radiotherapy [13], and abnormal Body Mass Index (BMI) values [4], also play an important role in predisposing patients to PP.

Intraoperative factors have been demonstrated to exert an influence on postoperative outcomes. These factors include surgical and anesthetic considerations such as duration of surgery or anesthesia. The depth of anesthesia and monitoring of vital signs are critical factors in postoperative outcomes in thoracic surgery patients [14]. The occurrence of intraoperative complications, in addition to the necessity for blood transfusions or high-volume crystalloid fluid transfusions, has been demonstrated to result in an increase in the incidence of postoperative PP as well [15,16]. These factors are associated with poorer postoperative results.

The choice of surgical technique, whether opting for an open thoracotomy or a minimally invasive procedure like Video-assisted thoracoscopic surgery (VATS) lobectomy, as well as the extent of lung resection, further determine the likelihood of postoperative complications [17,18]. The question of whether the surgical procedure leads to a lower or higher incidence of PP has undergone across the past years. Newer data show that VATS was associated with a lower incidence of PP than thoracotomy (THT) [18,19].

Post-operative care is equally important; the implementation of multimodal pain management strategies (e.g., Thoracic Epidural Analgesia (TEA)) is essential, as effective analgesia has been associated with a reduction in pulmonary complications, as is post-operative oral care [17,20]. In addition to the above, the encouragement of early mobilization, respiratory physiotherapy has and implementation of ERAS Protocols have the potential to reduce the incidence of postoperative pulmonary complications and accelerate recovery [20,21].

A comprehensive understanding of these risk factors is conducive to the development of targeted preventive strategies. The implementation of such strategies has the potential to improve clinical outcomes and reduce healthcare costs.

The German Thorax Register (GTR) plays a pivotal role in this process by systematically collecting perioperative data across multiple centers, thereby serving as a vital tool for quality assurance and benchmarking in thoracic surgery. The GTR’s pilot phase data provide a robust, evidence-based foundation for identifying risk factors specific to PP in anatomical lung resections, supporting continuous improvements in treatment protocols and overall patient care.

## 2. Materials and Methods

The study design is prospective and includes all patients undergoing anatomical lung resection at a single center, Cologne Merheim Hospital, University of Witten Herdecke in 2015. It was conducted as part of a one-year pilot phase of the GTR, with the aim of identifying risk factors for PP. Prior to participation, written informed consent was obtained from patients, and ethical approval for data analysis was obtained by the University Witten/Herdecke (Nr. 64/2014). The present study was conducted in accordance with the Declaration of Helsinki published 2013.

The inclusion and exclusion criteria were as follows: patients who underwent an anatomical lung resection at Cologne Merheim Hospital in 2015 (pneumonectomy, bilobectomy, lobectomy or segmentectomy) and provided written informed consent, with perioperative data documented in the registry through hospital discharge, were deemed eligible. Exclusion criteria encompassed non-anatomical lung resections, absence of written informed consent, procedures performed outside the 2015 study year or at other institutions, and cases with missing core data relevant to the primary endpoint (postoperative pneumonia) or key covariates.

Intraoperative ventilation was performed in accordance with the protocol outlined in the original registry-based thesis, employing pressure-controlled ventilation mode using Primus anesthesia machines (Drägerwerk AG, Lübeck, Germany). The one-lung ventilation strategy was predominantly facilitated via left-sided double-lumen tubes (86.1%).

The surgical procedures encompassed pneumonectomy, bilobectomy, lobectomy and segmentectomy, meticulously selected to ensure the standardization and comparability of the surgical interventions, non-anatomical resections and cases with missing data were excluded. The GTR encompasses a comprehensive set of preoperative, intraoperative and postoperative parameters, meticulously stored in an access database in a pseudonymized form. The preoperative parameters included the ASA classification, FEV1, CRP, White Blood cell count (WBC), smoking status and prior treatments. The intraoperative parameters obtained from the GTR included duration of surgery, the type of procedure, anesthesia method, one lung ventilation, fluid administration, blood transfusion and catecholamine therapy. The postoperative parameters included initial ward transfer (Intermediate Care (IMC)/Intensive Care Unit (ICU)), ventilation duration and postoperative complications.

Postoperative pneumonia cases were identified through a combination of registry documentation and ICD-10 discharge diagnoses provided by the hospital’s medical controlling department. The diagnosis was carried out by the attending staff. The period of follow-up for data capture concluded at hospital discharge. The term PP is defined in conjunction with clinical, laboratory and imaging findings [22].

The statistical analysis was performed with SPSS for Windows (V. 25.0). The descriptive statistics included mean (M), standard deviation (SD), and frequencies (f). The hypothesis testing was conducted with the Chi-squared test and the Fisher exact test. A significance level of <0.05 was employed. It should be noted that univariate analysis was the sole statistical method employed in this study. Due to the limited number of postoperative pneumonia events relative to the number of candidate predictors and incomplete documentation of some comorbidities and functional parameters, multivariable modeling was deliberately not performed in order to avoid overfitting and unstable estimates. Consequently, univariate analyses were employed, and the results are presented as associations rather than independent predictors. As the study was exploratory and hypothesis-generating, no formal adjustment for multiple statistical comparisons was performed.

## 3. Results

### 3.1. Descriptive Statistics

A total of *n* = 381 sufficiently completed patient datasets were included in the study, corresponding to *n* = 389 surgical procedures. The patient cohort had a mean age of 66.6 ± 11.4 years, with 59.6% male patients and an average BMI of 25.9 ± 4.5 kg/m^2^. The majority of patients were classified preoperatively to American Society of Anesthesiologists (ASA) category 2 (40.7%) or 3 (57.5%). Tumor surgery was the primary indication for surgery in 85.8% of cases. A positive smoking history (active smoker and/or long-term nicotine abuse) was documented in 86.6% of patients. Before the study period, 22% of patients had undergone radiotherapy, chemotherapy, or prior lung surgery.

Anesthesia induction was performed exclusively with sufentanil and propofol. Intraoperative ventilation was pressure-controlled (PCV mode) using a Primus anesthesia machine (Drägerwerk AG, Luebeck, Germany), with lung separation primarily achieved via left-sided double-lumen tubes (86.1%). The mean inspiratory oxygen fraction (FiO_2_) was 0.66 ± 0.14. Anesthesia maintenance primarily involved sufentanil (99.7%) and propofol (99.2%), with additional volatile anesthetics (sevoflurane or isoflurane) used in 29.6% of cases. The mean intraoperative volume of crystalloid infusion was 2584 mL ± 1643 mL (13.31 mL/kg/h ± 8.46 mL/kg/h). Hemodynamic stabilization required norepinephrine in 69% of patients, while 5.2% received blood products. Tranexamic acid was administered in 34.9% of cases.

The surgical procedures included lobectomies (64.3%), segmental resections (27.8%), bilobectomies (3.9%), and pneumonectomies (3.9%). Sleeve resections were performed in 15.7% of patients, and extended resections (angioplasty, pericardium/atrium, chest wall, diaphragm) were required in 22.3%. Surgical access was achieved via THT in 54.6% and VATS in 45.4%. The mean duration of surgery was 146.9 ± 52.7 min, with THT taking 164.2 ± 53.4 min and VATS procedures 125.9 ± 43.4 min. VATS was primarily performed for resections up to lobectomies, with mean durations of 163 ± 53.4 min for thoracotomy lobectomies and 132.8 ± 44 min for VATS lobectomies.

Perioperative monitoring included invasive blood pressure measurement in 97.4% of patients. A central venous catheter was placed in 36.7% of cases.

Postoperative analgesia included regional anesthesia in 43.3% of patients, predominantly for planned THTs. Paravertebral blocks (PVBs) (41.5%) were the primary technique, while TEA was used in only 1.8% of cases. Primary postoperative transfer occurred to IMC (66.1%) or ICU (29.1%) ward, with only 4.7% of patients being directly transferred to a general ward after post-anesthesia care. Determinants for postoperative treatment in the ICU are displayed in Table 1.

The average ICU stay was 3.4 ± 7.1 days, with 19.8% of ICU patients requiring postoperative intubation. The mean duration of invasive ventilation was 33.9 ± 129.8 h, though due to non-parametric distribution, the median ventilation time was 16 h (IQR = 56), and the median ICU stay was 1 day (IQR = 1). The in-hospital mortality rate was 2.6%. The majority of patients (97.4%) were discharged after an average hospital stay of 14.7 ± 9.1 days.

PP was diagnosed in 15.2% of patients, with a mortality rate of 10.3% among pneumonia cases. In contrast, mortality among patients without PP was 1.2%. Pneumonia was significantly associated with mortality (*p* = 0.01). Additional significant determinants of perioperative mortality included surgery duration > 180 min (*p* = 0.016), extended chest wall resection (*p* = 0.001), postoperative ICU admission with intubation (*p* = 0.001), and mechanical ventilation > 24 h (*p* = 0.006).

### 3.2. Analysis of the Risk Factors for Postoperative Pneumonia

#### 3.2.1. Preoperative Factors

**Figure 1:**
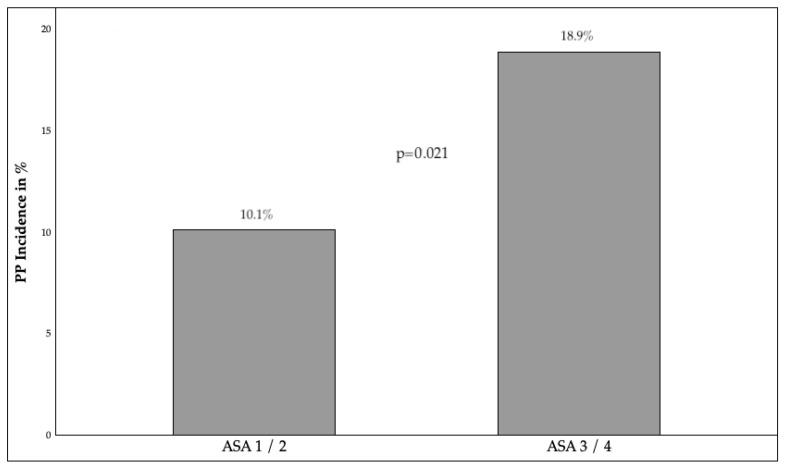
ASA classification and PP incidence; ASA = American Society of Anesthesiologists Score. PP = Postoperative Pneumonia. *p* < 0.05 indicates statistical significance.

The findings of this study demonstrate that patient age, gender and BMI are not predictors of the development of PP. Furthermore, the study shows that there is no correlation between the rate of PP and the presence of preoperative radiotherapy or chemotherapy. However, the study does demonstrate that, with an ASA classification ≥ 3, the risk of pneumonia increases significantly. The study also shows that smoking history is not a statistically significant factor. Figure 1 shows the association between ASA Classification and PP Incidence.

**Figure 2:**
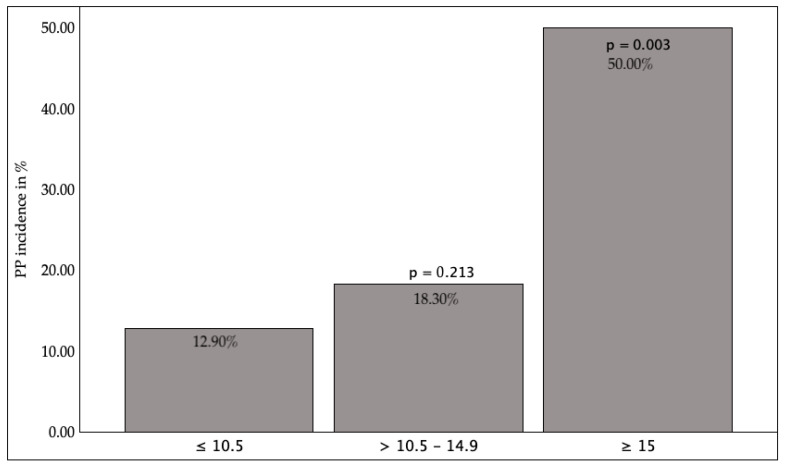
WBC and PP incidence. WBC = White Blood Cell Count. PP = Postoperative Pneumonia. *p* < 0.05 indicates statistical significance compared to the normal value.

In comparison with the standard value (CRP ≤ 3 mg/L), elevated preoperative CRP values, despite a clear increase in the incidence of PP, did not demonstrate any significant disparities. However, to improve the discriminatory power of the analysis, the cohort was subsequently stratified into two clinically meaningful subgroups: CRP < 20 mg/L and CRP ≥ 20 mg/L. Utilizing this revised grouping, a significant association was identified between elevated CRP levels and the occurrence of PP. In the laboratory data, both elevated CRP above 20 mg/L (*p* = 0.004) and WBC count above 15,000 µL (*p* = 0.003) were identified as significant predictors of the probability of developing PP. Figure 2 illustrates the association between WBC levels and the occurrence of PP.

**Figure 3:**
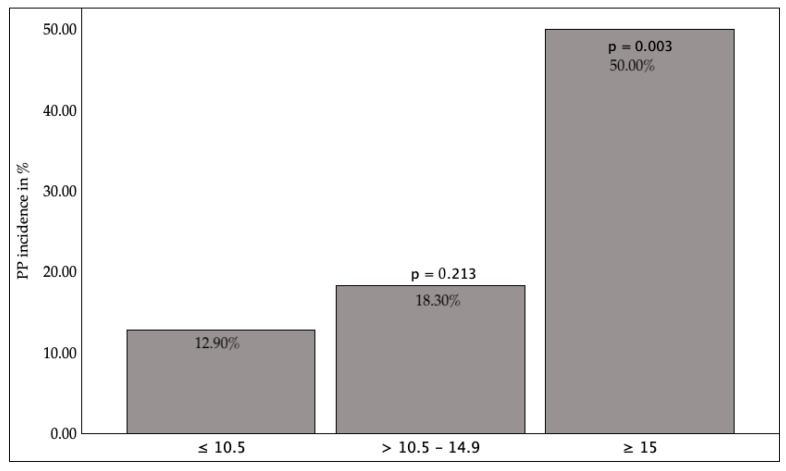
FEV1 preoperative and PP incidence. FEV1 = Forced expiratory volume in one second. *p* < 0.05 indicates statistical significance.

**Figure 4:**
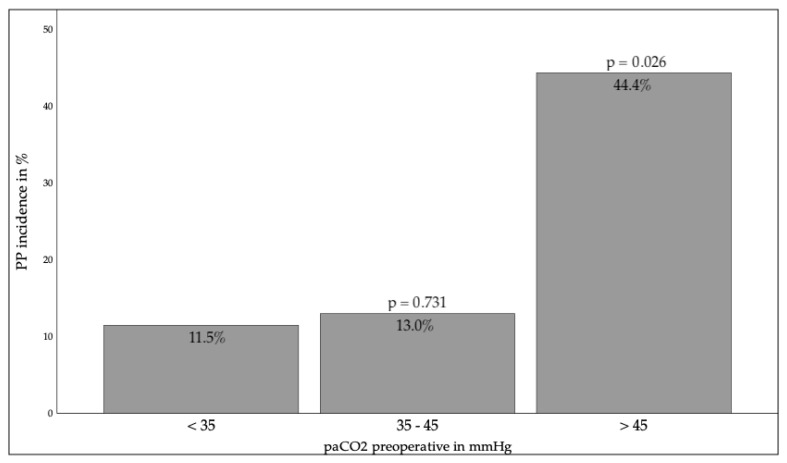
paCO_2_ preoperative and PP incidence. paCO_2_ = Partial Pressure of Carbon Dioxide PP = Postoperative Pneumonia mmHg = millimeter(s) of mercury *p* < 0.05 indicates statistical significance compared to the standard value.

In relation to the lung function test, oxygenation disorders (diffusion capacity, KCO, partial pressure of Oxygen (paO_2_)) demonstrated no influence; however, ventilation disorders (FEV1, partial pressure of CO_2_ (paCO_2_)) (Figure 3 and Figure 4) were associated with a significantly increased pneumonia rate. The risk was found to be increased in cases where the FEV1 was less than 50% of the target value (*p* = 0.004) or where the paCO_2_ was greater than 45 mmHg (*p* = 0.026).

In the study, 15% of patients who developed PP had never smoked, 17.2% were former smokers with an abstinence period of over three months, and 15.2% were smokers or had an abstinence period of less than three months. The study revealed no statistically significant differences in the incidence of PP between smokers who had abstained for more than three months and those who had been smokers or had abstained for less than three months (*p* = 1.000).

#### 3.2.2. Surgical Factors

**Figure 5:**
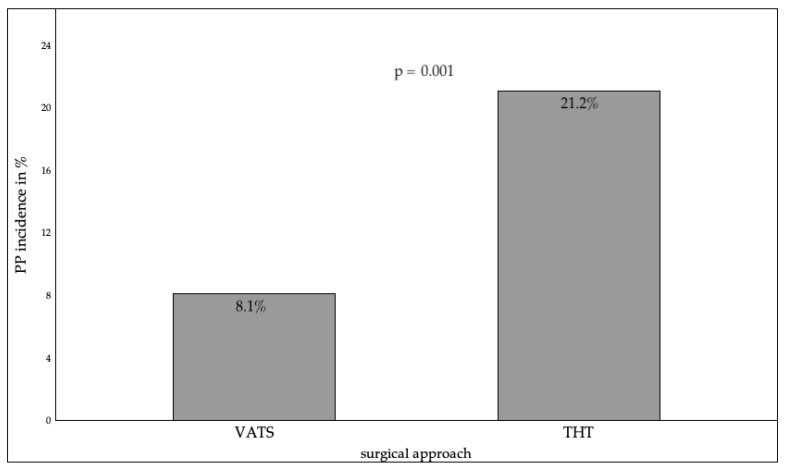
Operative access route and PP incidence. VATS = Video-assisted thoracoscopic surgery THT = Thoracotomy. *p* < 0.05 indicates statistical significance.

The surgical approach demonstrated a significant correlation with the occurrence of PP, with THT exhibiting a substantially elevated risk in comparison to VATS (21.2% vs. 8.1%, *p* = 0.001). However, no substantial discrepancy in the incidence of PP was observed between left and right THT (*p* = 0.252). Furthermore, the duration of surgery exhibited a discernible correlation with the risk of PP. While an initial increase in the incidence of PP was observed for procedures lasting more than 90 min, statistical significance was only reached for procedures lasting more than 180 min (*p* = 0.002). To further analyze this effect, surgery duration was divided into three groups, with no significant difference found between procedures lasting ≤90 min (4.3%) and those lasting between 91 and 180 min (13.4%, *p* = 0.089). Operating times exceeding 180 min were significantly associated with a higher incidence of PP (30.8% compared to the 91–180 min group, *p* = 0.002). A detailed analysis revealed a progressive increase in PP risk with longer operating times, ranging from 6.3% for operations ≤ 60 min to 40.0% for operations lasting 241–270 min (*p* = 0.018). The surgical approach, along with the prevalence of PP, has been illustrated in Figure 5.

#### 3.2.3. Anesthesiologic Factors

The choice of anesthetic agent, the double lumen tube (left/right) and the additional use of a regional anesthesiologic procedure (TEA or PVB) had no significant influence on the pneumonia rate. Similarly, there was no difference in this respect between continuous epidural anesthesia and PVB in the single-shot procedure.

The regional anesthesiologic procedures were used almost exclusively for THTs in the study population. A further evaluation of the regional anesthesiologic procedures in the subgroup of THT was necessary. However, no significant difference in the incidence of PP was found here.

A plethora of anesthesiologic factors, including the mean FiO_2_, the intraoperative infusion volume, catecholamine therapy and the requirement for blood transfusion, were not found to be associated with the development of PP. Exact results are shown in Table 2.

#### 3.2.4. Postoperative Factors

A total of 27% of the patients primarily admitted to the ICU postoperatively demonstrated PP. In comparison, it was only 10.4% for patients transferred to the IMC or regular ward (*p* < 0.001). Significantly increased odds of PP were found for ICU stay >2 days (*p* = 0.001), postoperative transfer of an intubated patient to the ICU (*p* = 0.002), and postoperative ventilation >24 h (*p* = 0.001).

## 4. Discussion

PP is a significant complication following thoracic surgery, associated with increased morbidity, mortality, and prolonged hospital stays. The present study identified a 15.2% incidence of PP among patients undergoing anatomical lung resections. The following preoperative risk factors were identified as being of key significance: an ASA classification of ≥3, elevated CRP levels (≥20 mg/L), and leukocytosis (WBC ≥ 15,000/µL). Intraoperative risk factors were found to be associated with THT and prolonged surgery duration (>180 min), while postoperative predictors included ICU admission and mechanical ventilation > 24 h. These findings are consistent with the current literature, which emphasizes the importance of thorough preoperative risk assessment and optimized perioperative management.

The statistical strategy of this work must be interpreted in light of its exploratory, registry-based nature. The present analysis was constrained to univariate associations utilizing predefined, clinically plausible thresholds, with multivariable modeling and adjustment for multiple testing not included. Consequently, it is not possible to assert that the identified variables are independent predictors of PP, and a proportion of the statistically significant findings may be attributable to false positive error. Furthermore, it is important to note that several of the observed risk factors are likely to be interrelated rather than independent. Inflammatory markers such as CRP and WBC both reflect systemic inflammation, and higher ASA class and impaired FEV1 tend to co-occur with more complex perioperative courses, including ICU admission and prolonged mechanical ventilation. In this context, it is imperative to interpret ICU stay and prolonged mechanical ventilation as markers of severity along the clinical pathway from pre-existing vulnerability to postoperative complications, rather than as isolated upstream causal risk factors. Consequently, the present data describe clusters of risk rather than disentangling the individual contributions of each variable.

The findings of this study corroborate those of previous research, underscoring the significance of the ASA classification as a risk factor for PP. As demonstrated by Leporta et al. and Agostini et al., an ASA score of 3 or more has been shown to result in a significant increase in the likelihood of postoperative complications, including pneumonia, particularly in non-cardiothoracic surgery [4,23]. A study encompassing a sample population of over two million patients has reiterated the value of the ASA classification system in predicting significant postoperative complications, including PP. This classification system is regarded as an independent risk factor for the development of postoperative complications and mortality [24]. Further factors, including BMI, have been demonstrated to exert an influence on the incidence of PP [25]; however, these were not the focus of analysis in the present study.

The correlation between heightened CRP levels and postoperative complications has been documented in previous studies [12,26]. Okada et al. reported that a preoperative CRP ≥ 20 mg/L is associated with increased postoperative mortality following lung resection, a finding that aligns with the results of the present study [12]. It is important to note that an elevated CRP level may be an indication of an undiagnosed preexisting infection, including subclinical pneumonia that was present at the time of surgery. This may have the effect of influencing the postoperative complications. Furthermore, the relationship between leukocytosis and PP risk is supported by studies indicating that preoperative leukocytosis (>11,000/µL) may predict inflammatory complications, not only in thoracic surgery but also in cardiac surgical patients [27].

With regard to the question of smoking as a risk factor, the present study did not establish a statistically significant correlation between short-term smoking cessation (up to three months before surgery) and reduced PP incidence. This finding is in contrast with the results of earlier research, which indicated that longer periods of smoking cessation were associated with a reduced risk of postoperative complications [4,28,29,30]. Takenaka et al. proposed that cessation for a period of at least one month may mitigate the risk of PP, while Zhang et al. demonstrated that abstinence exceeding one month is particularly beneficial in patients undergoing VATS. The absence of association in the present cohort may be attributable to the limited duration of smoking cessation, as well as potential confounding factors such as socioeconomic status and cumulative tobacco exposure.

In addition to cumulative tobacco exposure, the presence of COPD appears to be a pivotal factor in the development of PP [25,31]. This was not examined in the present study, but it is one of the most common risk factors for PP in thoracic surgery [25]. However, the absence of an association between COPD and PP in the present study may be attributable to a number of factors. These include the lack of a systematic assessment of COPD status, the potential overlap with other respiratory risk factors, and methodological limitations inherent to the single-center study design.

In this cohort, the THT was associated with higher PP rates, which may be attributed to greater tissue trauma, increased inflammation, and postoperative pain in comparison with minimally invasive VATS. VATS procedures have been demonstrated to be associated with reduced operation durations and the potential for superior outcomes. This finding is consistent with the results of a nationwide Swedish study by Al-Ameri et al., which reported a significantly higher incidence of PP in THT patients (5.5% vs. 0.6%) and a higher 30-day survival rate in VATS patients (VATS 0.3% vs. 0.7% in THT) [32]. These findings are consistent with those of a meta-analysis conducted by Francis et al., which indicated a reduced risk for post-pneumonectomy PP in patients who underwent VATS [18]. VATS also demonstrated enhanced pulmonary function [33]. In a 2018 study by Xie et al., it was demonstrated that the VATS technique reduced operation time and the necessity for blood transfusions [34]. Moreover, the minimally invasive nature of the VATS procedure has been demonstrated to be associated with a reduced level of pain and a decreased length of hospitalization [18,35]. In the preceding three decades, a considerable transition has been observed from the utilization of THT to that of VATS. It is imperative to consider this when comparing the study. Despite the distribution between THT and VATS in 2015 being 54% to 45%, as shown in this study, it is hypothesized that the ratio is 24% to 70% and 6% (other surgical possibilities) in the current context [36].

Furthermore, the increasing establishment of minimally invasive mediastinal staging procedures (EBUS-TBNA) over mediastinoscopy could improve preoperative selection and thus indirectly reduce perioperative risks, including PP. A recent systematic review has demonstrated that EBUS-TBNA has high sensitivity and specificity while also offering a more favorable safety and cost profile compared to mediastinoscopy [37].

The study demonstrates a strong association between mechanical ventilation for over 24 h and an increased risk of PP, as well as a significant association between intubation duration and the development of PP, which aligns with the recent literature [38]. Extubation should ideally take place at an early stage and in conjunction with lung-protective ventilation strategies [39].

ERAS protocols may have also a positive impact on the rate of hospitalization. It also highlights the importance of regional anesthesia, particularly in open surgery, to improve recovery after lung resection [20], but were not specifically analyzed in this study.

The present study demonstrates that procedures lasting over 180 min are associated with a marked elevation in the risk of PP. It is therefore recommended that surgical times be reduced and introspective ventilation techniques employed [38,40]. Wang et al. identified a surgery duration of more than 3 h as a risk factor for developing PP in lung cancer patients [41].

The patients in this study who developed a PP also exhibited a significantly longer duration of stay in the ICU when compared to patients without PP. This finding is in alignment with clinical practice, which shows that the existence of a PP is indicative of a critical condition that necessitates treatment on an ICU ward.

Notwithstanding the valuable statistical results obtained, this study is not without limitations. Firstly, the study is limited in its generalizability due to its single-center design. Secondly, the incomplete data on specific comorbidities and diagnostic criteria may affect the precision of risk evaluations. The dearth of granular data regarding specific comorbidities and functional status, beyond the scope of the ASA classification, hindered comprehensive analyses. Moreover, the precise temporal sequencing and diagnostic precision of PP events remained challenging to determine. The absence of systematic microbiological data, such as preoperative sputum colonization with specific pathogens, precluded the analysis of these data as potential modifiable factors. Thirdly, it should be noted that only univariate analyses were performed, without formal correction for multiple comparisons. This approach was selected a priori, given the limited number of PP events and the relatively extensive set of candidate variables. Additionally, the aggregate nature of comorbidity coding would have rendered multivariable models unstable and potentially misleading. Consequently, the observed associations should be interpreted as hypothesis-generating rather than as proof of independent predictors, and an inflation of false positive error cannot be excluded. A further limitation is the absence of granular intraoperative ventilatory data in the 2015 GTR pilot dataset. However, the analysis was unable to consider the impact of PEEP levels and tidal volumes normalized to ideal body weight (mL/IBW) on postoperative pulmonary outcomes, despite the relevance of these parameters in such cases.

In order to confirm the findings of this study, refining risk stratification models and identifying truly independent, modifiable predictors of PP; adequately powered multicenter studies with standardized PP definitions; detailed clinical phenotyping (including microbiology); precise intraoperative ventilator settings; and prespecified multivariable modeling are required. The further development of thoracic surgery registries, such as the GTR, will be central to this endeavor.

## 5. Conclusions

Postoperative pneumonia following anatomical lung resection remains prevalent and has significant clinical implications. In the present cohort, elevated preoperative risk (ASA classification ≥ 3, CRP ≥ 20 mg/L, WBC ≥ 15,000/µL) was associated with increased PP, while intraoperative factors (THT over VATS and operative time > 180 min) and postoperative factors (mechanical ventilation > 24 h and ICU admission) further amplified risk. Conversely, VATS appeared to offer a protective benefit, supporting a minimally invasive, time-efficient surgical strategy where feasible. These findings argue for a structured perioperative pathway that combines rigorous preoperative optimization (e.g., control of inflammation, smoking cessation), preference for VATS, adherence to lung-protective ventilation with early extubation, and integration into ERAS programs. Despite being derived from a single-center 2015 cohort, the signals are biologically plausible and actionable. Multicenter, prospective validation with standardized PP definitions should refine risk prediction and quantify the benefit of targeted interventions.

## Figures and Tables

**Table 1:** Determinants for postoperative treatment in the ICU; ICU column: Percentage of patients within each subgroup who were treated in the ICU.

ASA	ICU	*p*-Value
1–2	22.2%	0.012
3–4	34.2%	
**Surgery**	**ICU**	***p*** **-Value**
Pneumonectomy	93.3%	<0.001
Bilobectomy Lobectomy Segment resection	76.9% 29.8% 12.8%	<0.001 <0.001 <0.001
**Operative access route**	**ICU**	***p*** **-Value**
VATS	9.2%	<0.001
THT	45.7%	<0.001

ASA = American Society of Anesthesiologists Score; ICU = Intensive Care Unit; *p* < 0.05 indicates statistical significance; VATS = Video-assisted thoracoscopic surgery; THT = Thoracotomy.

**Table 2:** Postoperative pneumonia (%): Proportion of patients with postoperative pneumonia per subgroup.

Anesthetic Form	PP	*p*-Value
TIVA	17.2%	0.252
balanced anesthesia pure general anesthesia combination process (+TEA/PVB) TEA PVB (Single Shot)	12.6% 13.0% 18.2% 14.3% 18.4%	0.252 0.195 0.195 0.785 0.785
**Anesthetic form (THT only)**	**PP**	***p*** **-Value**
pure general anesthesia	43.2%	0.862
combination process (+TEA/PVB)	56.8%	
**Double lumen tube**	**PP**	***p*** **-Value**
Left	14.9%	0.803
right	17.6%	

TIVA = Total intravenous anesthesia; TEA = Thoracic Epidural Analgesia; PVB = Paravertebral block; PP = Postoperative Pneumonia; THT = Thoracotomy; *p* < 0.05 indicates statistical significance.

## Data Availability

The data presented in this study are available upon request from the corresponding author.

## Abbreviations

ASA	American Society of Anesthesiologists
BMI	Body Mass Index
COPD	Chronic Obstructive Pulmonary Disease
CRP	C-Reactive Protein
DLCO	Diffusion Capacity of the Lung for Carbon monoxide
EBUS-TBNA	Endobronchial ultrasound-guided transbronchial needle aspiration
ERAS:	Enhanced Recovery After Surgery
FEV1	Forced Expiratory Volume in one second
FVC	Forced Vital Capacity
GTR	German Thorax Register
ICU	Intensive Care Unit
IMC	Intermediate Care Unit
KCO	Carbon Monoxide Transfer Coefficient
PCV	Pressure-Controlled Ventilation
PP	Postoperative Pneumonia
PPCs	Postoperative Pulmonary Complications
PVB	Paravertebral Block
SD	Standard Deviation
SPSS	Statistical Package for the Social Sciences
THT	Thoracotomy
TNM	Tumor, Node, Metastasis
TEA	Thoracic Epidural Analgesia
UICC	Union for International Cancer Control
VATS	Video-Assisted Thoracoscopic Surgery
WBC	White Blood Cell Count
